# Changing approaches to green infrastructure design: from modernism to the future: Warsaw case study

**DOI:** 10.1057/s41289-023-00220-5

**Published:** 2023-05-17

**Authors:** Krystyna Solarek, Anna Domaradzka, Justyna Zdunek-Wielgołaska

**Affiliations:** 1 Faculty of Architecture, Warsaw University of Technology, ul. Koszykowa 55, 00-659 Warsaw, Poland; 2 Institute for Social Studies, University of Warsaw, ul. Stawki 3/5, 00-183 Warsaw, Poland

**Keywords:** Modernism, Urban planning, Green infrastructure, Green wedge, Warsaw

## Abstract

International modernism has brought new solutions to urban greenery, primarily aimed at improving the living conditions of the inhabitants. Currently, much more is expected of green areas, and at the same time, various impacts influence their modifications. The article focusses on changing approaches to the issue of green wedges design resulting from the socio-political transformation and the changes in approaches to city planning priorities. We selected Warsaw for detailed research, where the evolution of this approach can be clearly observed. The study showed the changes in the size, layout and function of one of the most important green areas and residents’ expectations. The formulated conclusions and threads may be helpful in spatial policy, spatial planning and urban design in various cities with an open areas structure similar to Warsaw.

## Introduction

Modernism as a movement that arose in response to the ecological and social inefficiency of nineteenth-century industrial cities brought greenery to them on a large scale—green housing estates, greenways, green systems—belts, lanes and wedges. Greenery was to structure cities and regions, separate functional zones, isolate them from nuisance, constitute land reserves and serve recreation. Greenness and open space systems were therefore the basic organising structure, and even a planning tool, whatever the scale and the form (Walmsley 1995; Fung and Conway 2007; Fábos and Ryan 2004). The prevailing view was that green belts, most often in the form of rings or linear parks, were a way to bring order to spread-out suburbs and rebuilt inner-city neighbourhoods, and at the same time to preserve natural habitats (Walmsley 1995; Amati and Taylor 2010). For many years and in many cities around the world, the issue of their changing functions and maintenance problems has been raised (Amati and Taylor 2011; Amati and Yokohari 2007; Terry et al. 2006; Breiling and Ruland 2008; Ali 2008).

The specific approach to shaping greenery had a particular impact on European cities that were rebuilt after the large-scale destruction of the Second World War, such as Berlin, Dresden, Kyiv, Szczecin and Warsaw; this also applied to numerous smaller towns. Following the then dominant views on urban planning, including the guidelines of the Charter of Athens, the pre-war urban structure was not recreated, but new assumptions were shaped (Musiaka et al. 2021; Lubocka-Hoffmann 2008). According to Le Corbusier’s ideas of ‘functionalism’, open areas had a huge role to play in them (Mehaffy et al. 2018). Those of the greatest importance usually formed a band or band-concentric system, creating green wedges (Lemes de Oliveira 2017; Ryńska and Solarek 2018). In the countries of the former Eastern Bloc, rebuilt after the war, these generously delineating green areas assumed enormous dimensions. They were often detached from the natural resources and accompanied large communication arteries (Solarek 2017). A similar approach characterised new cities and districts built in the mid-twentieth century, such as Harlow, Milton Keynes, Bijlmermeer in Amsterdam, planned in accordance with the guidelines of the 1933 Charter of Athens. Suchlike phenomena also occurred outside Europe, for example, in Asian cities (Yokohari et al. 2000). There has been a tendency to focus on the passive green elements rather than on a positive, urban form-making ones. In some cases, green wedges became vast ‘nobody’s’ areas, difficult to maintain and hindering the formation of coherent neighbourhoods (Walmsley 1995). The spatial structures shaped in this way tend to be troublesome today, and green wedges are modified in various ways.

Thus, the approach to shaping the relationship between built-up and open areas determined the development of many cities and largely depended on whether the priorities of early modernism or the modern avant-garde movement were followed. Both visions were a manifestation of modernism, putting them on opposite poles: the cultural and progressive trends, or also: the traditionalism and avant-garde (Kononowicz 2007; Walmsley 1995). The avant-garde trend—with the idea of functional city—proposed by the Charter of Athens mostly influenced the development of European cities, additionally stimulated by socio-political conditions, especially in the countries of the former Eastern Bloc, including Poland.

After several years, the excessive extent of green areas, the failure to respect the conditions arising from the issue of land ownership, the principles of land management and government, as well as the delay in planning work concerning the ongoing investment processes, have led to significant modifications of the original modernist concepts, The significant decline in the area and the number of green spaces that grew so significantly during the period of socialism and late modernism was due to the weak legislative framework, land restitution and ownership conflicts (Badiu et al. 2019).

While one of the most interesting issues is how the morphology of cities was approached according to these two particular trends of modernism, an equally interesting problem is the evolving change in the green areas’ functions. In the Functional City concept, leisure was an essential element and greenery was the key to its implementation (Le Corbusier 1973; Mehaffy et al. 2018). In the former Eastern Bloc countries seemingly freely shaped green areas, often surrounding loosely located buildings, were strictly subordinated to top-down functions, determined based on rational premises, established in the form of regulatory plans, technocratic guidelines, although not without a thorough examination of the existing physiographic conditions. Here and there it turned into employee gardens, sometimes squares or lanes by roads, often into wastelands. The emphasis was placed on the needs and goals of the group above the needs of everyone; therefore, large leisure parks were also created here (Solarek 2017).

Today, it is necessary to consider the fact that green area systems, sometimes maintained in cities for decades, have lost some of their original functions and image of late. They no longer separate the zones of different use and purpose because we have already negated the vision of a Functional City, and they do not serve to provide fresh air from the suburbs, because the suburbs have ceased to be its reservoir. Developers are waiting for the possibility of building on them because land prices in cities are constantly growing. The pressure to intensify development is increasing and the city authorities are also succumbing to it. And, at the same time, the new functions of green areas are of growing interest.

Although it still remains a prevalent view that green belts and other systemically shaped green areas have the urban separator function (White et al. 2010), serve the urban sprawl containment (Ali 2008) and recreation (Terry et al. 2006), some new concepts are being raised. Hebbert (2008) emphasises the role of greenery in the climate change mitigation and adaptation and generally, it is becoming more and more common to treat green belts as elements of green infrastructure (Amati and Taylor 2010). Urban green infrastructure as a network of natural and semi-natural elements provides ecological, economic and social benefits for humans and other species. It is increasingly often treated as an important element promoting sustainability—a societal goal based on the same three dimensions (Wolch et al. 2017).

But a sustainable approach requires taking into account many aspects in city planning, therefore ecological solutions should be introduced, which are based on balancing the expected economic effects with the growing environmental and social needs (Jaszczak et al. 2020). ‘The public perception of the green belt is essential to generate the political will for successful implementation and management. If neither planners nor the public perceive value in the green belt, other visions will surely supplant the green belt’ (Amadi and Taylor 2010, p. 150). This means that great emphasis should be placed on increasing the awareness and knowledge of planners, but also meeting social expectations in relation to urban greenery.

Literature helpful in combining various topics related to planning green systems has a gap in the evolutionary approach to greenery systems at the turn of the twentieth and twenty-first centuries. It is difficult to find literature in which the problem of transforming the spatial and functional structure of green areas is related to the analysis of social preferences and the resulting indications for planning practice. So when simultaneously focussing on the planning-morphological and social issues, one can pose a few important questions, concerning Warsaw and other cities: (1) How and why has the form of the green wedges changed from the era of modernism to the present day? (2) How have the functions of green areas, systematically planned in the times of modernism, changed over time? (3) What are the current social expectations of Warsaw residents regarding green areas? To answer the above questions, we conducted a study in Warsaw analysing the green areas system and a selected green wedge—formerly planned and actual layout and their functions. We also examined the expectations of Warsaw residents regarding urban greenery and ways of recreation.

## Materials and methods

**Fig. 1:**
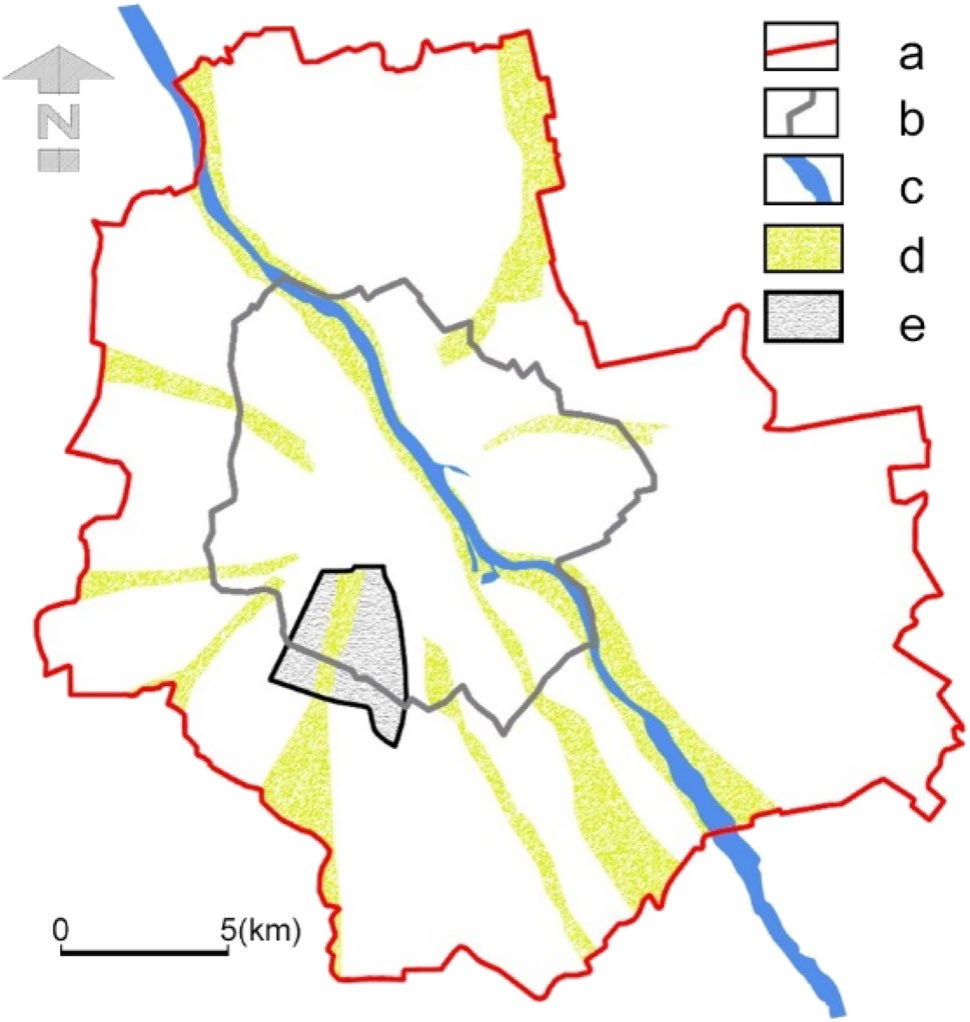
Location of the research area in Warsaw. (a) Current city borders, (b) city borders in 1930, (c) Vistula River, (d) ventilation corridors and (e) studied area. *Source* own study

In the first stage of the study, we analysed the literature on the subject to identify aspects related to the contemporary and historical approach to shaping urban green areas. Particular attention was paid to the chosen green wedge of Warsaw spatial structure as an example to analyse the changes taking place over time. The area was selected for research because it has undergone multiple spatial transformations, and it is also home to facilities and areas important to residents (Fig. 1). We analysed selected projects and spatial development plans in Warsaw drawn up in the twentieth century and at the beginning of the twenty-first century—their text and drawing parts. We also researched their social and political context as well as relation to the urban planning trends.

**Fig. 2:**
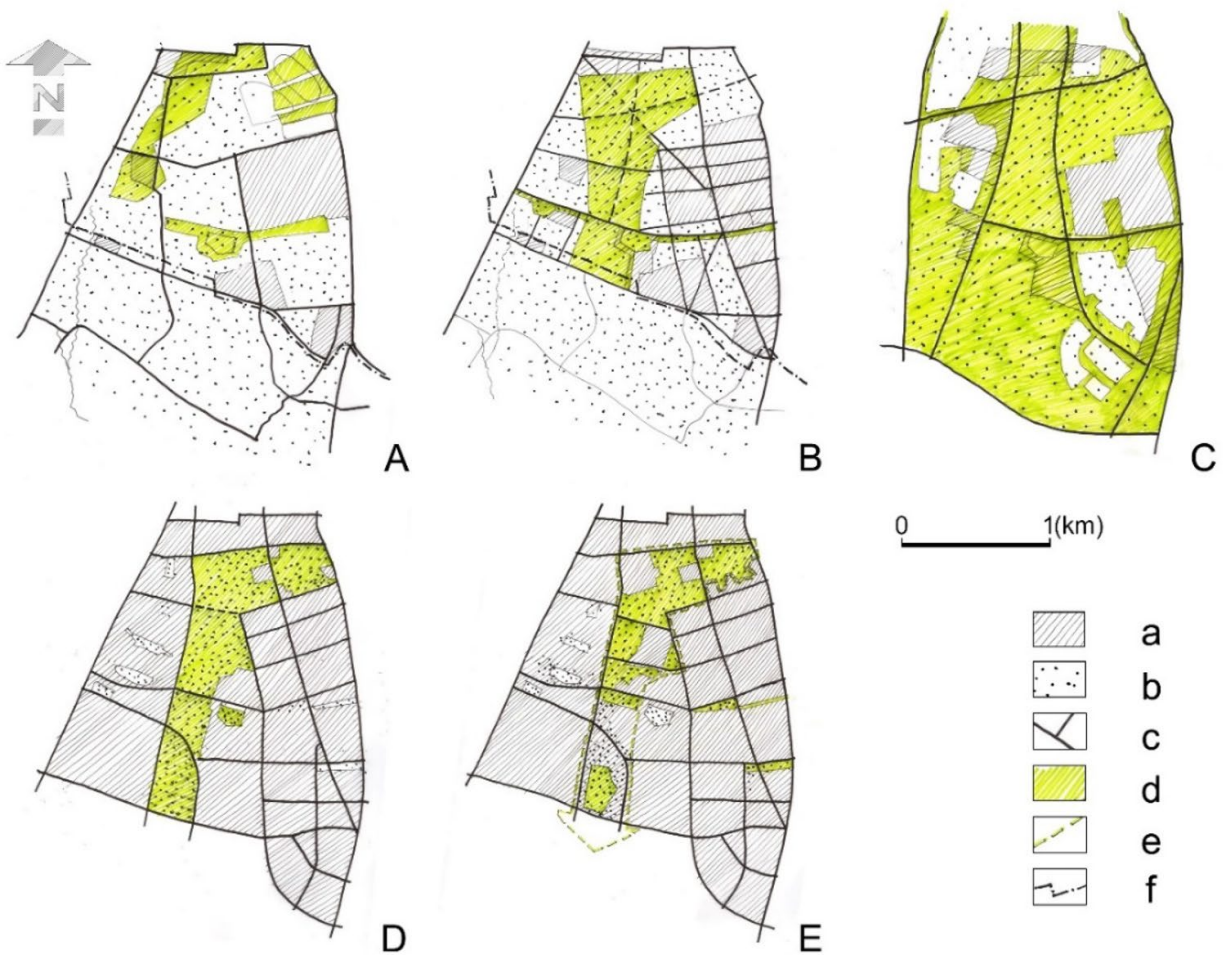
Diagrams showing visions of shaping urban green areas (date before slash) against the background of actual land cover during the development of the studied urban plans (date after slash). **A** 1916/1924, **B** 1931/1939, **C** 1945/1945, **D** 1982/2006 and **E** 2006/2021; signs: (a) built-up areas, (b) open areas, (c) major existing and planned roads, (d) planned urban green areas, € boundary of the supply and air exchange corridor and (f) administrative border of Warsaw from the beginning of the twentieth century (diagrams **A** and **B**). *Source* own elaboration

A simplified state of land cover has been compiled on historical and current maps, distinguishing built-up areas and open areas and the layout of green areas planned at that time. We integrated different data sets—land cover forms and planned urban structures, based on the cartographic maps from 1916 to 1982 (Table 1). Additionally, digital databases were used based on the following portals: ongeo.pl, geoportal360.pl, mapa.geoportal.gov.pl, and warszawa1939.pl. The diagrams (Fig. 2) show the arrangements of the plans that were prepared for the entire City of Warsaw within the borders valid as at the date of their preparation, related to the research area.

**Table 1:** Cartographic materials used in the data collection process

Year	Name of the plan, publisher, place of publication	Scale
1916	Preliminary sketch of the Regulatory Plan, Association of Architects, Warsaw	1:10,000
1924	Plan of the Capital City of Warsaw. Military Geographical Institute, Warsaw	1: 20,000
1930 (1931)	General Development Plan for the Capital City of Warsaw, Municipal office of the Capital City of Warsaw, Warsaw	1:10,000
1933	Plan of the Capital City of Warsaw, Municipal office of the Capital City of Warsaw, Warsaw	1: 25,000
1935	Plan of the Capital City of Warsaw, Cartographic Institute name of E. Romer. Publisher Książnica-Atlas S.A., Lviv	1:25,000
1939	Plan of the Capital City of Warsaw and the surrounding area, Warsaw City Board, City Planning Department, Warsaw	1:10,000
1941	Plan of Warsaw, Polnischer Verlag GmbH, Warsaw	1: 20,000
1945	Plan of the Capital City of Warsaw, Warsaw development concept, Warsaw Reconstruction Office, Warsaw	1:10,000
1948	Plan of the Capital City of Warsaw, Military Press, Warsaw	1: 25,000
1982	Perspective General Plan of Spatial Development of Warsaw, Warsaw Development Planning Office, Warsaw	1:10,000
2006	Study of the Conditions and Directions of Spatial Development of the Capital City of Warsaw, municipality of the Capital City of Warsaw, Warsaw	1:10,000

For the selected trends and time periods, we conducted an analysis of the applied urban design solutions, relating them to the then vision of shaping cities, as well as to the planning and political conditions. We also described the functions of open areas in the studied periods and the functions assigned to the green areas in the surveyed urban projects.

Next, we described and studied the social expectations of Warsaw residents regarding green areas. When making compilations and comparisons, we relied on the government census from 2002 (Spis Powszechny 2002), studies conducted by the City of Warsaw (Barometr Warszawski 2010–2021), Husqvarna’s Global Garden study from 2012, as well as online poll by YouGov from March 2021. Based on the recent study by the City of Warsaw Office ‘Quality of life of Warsaw districts’ residents’ from November 2019, we also calculated the correlation between residents' satisfaction with green areas and their social status. We analysed the data on three levels: representative sample of Warsaw residents (*N* = 9720), representative sample of Mokotów district residents (*N* = 540) and the subsample of residents from 5 selected MSIs (municipal unit of the information system, *N* = 380) covering the area of the chosen green wedge—‘Mokotów’s southern air exchange corridor’. Period of living in the area was calculated in years based on the question “Since which year do you live in this district”, which was then recoded into 5 categories: 5 years or less, 6–15 years, 16–25 years, 26–35 years and over 35 years. Education level was quantified based on the question about last completed level of education with 8 level scale, where 1 is incomplete primary education and 8 is higher (tertiary) education. Education level was not recoded.

The results obtained enabled us to identify the most important challenges for planning greenery systems and to answer our initial questions. It also contributed to the discussion concerning the possible approach to the contemporary shaping of green belts in Warsaw and other cities, where extensive green areas were introduced in accordance with the guidelines of the Charter of Athens.

## Results

### The evolution of Warsaw’s modern greenery system: an example of the southern green wedge

The conducted research made it possible to identify the main differences in the approach to urban greenery planning. Each diagram (Fig. 2A–E) showing the surface area and planned greenery in a synthetic way represents a separate urban trend. The results of the analyses are shown in graphical (Fig. 2) and tabular (Table 2) forms. This table presents not only the evolution of the spatial changes but also their political background as well as dominant social expectations and functions of the investigated open space at the time of adopting successive plans.

**Table 2:** Analysis of the approach to planning the southern green wedge in relation to individual urban and socio-political trends

The year in which the plan was created/the year of registration of the state of land cover The name of the plan	Selected legal and political conditions in spatial planning of Warsaw	The approach to designing urban structures	Dominant social expectations	Impact of the plan on greenery in the studied area (reference to diagram number in Fig, 2)	The real actual functions of the investigated open space at the time of adopting/presenting the plans
**1916/1924** Preliminary sketch of the Regulatory Plan	Just before Poland regained independence, a modern sketch plan of the city was prepared	Improving living conditions as **a sign of modernity** The sanitary role of greenery was noticed, large-scale urban layouts were applied, parks and avenues were planned	– Improvement of housing conditions (hygienic and health conditions)		– Airport – Horse racing track – Military areas – Farmlands – Wastelands
**1931/1939** General Development Plan for the Capital City of Warsaw	New legal provisions were created in the interwar period of free Poland, including construction law and public institutions. Despite the small share of municipal land in Warsaw, various public areas, including parks, were planned	**Early modernism** (a cultural trend), sublime urban composition based on the axes was applied. The role of greenery in improving air quality has been noticed; formal, composed green areas—the concept of a green wedge, formal parks and public amenities were planned	– The need for new recreational areas, public functions, as well as symbolic and functional evidence of political and economic recovery		– Open public areas, prepared for implementing the significant public investments (Piłsudski District) – Wastelands
**1945/1945** Plan of the Capital City of Warsaw. Warsaw development concept	Post-war damage. Communalisation of land, socialist planning, various approaches to rebuilding Warsaw with most votes in favour of building a new functional city	**Late modernism**, progressive trend. Implementation of strict CIAM guidelines—loose buildings among the greenery. Ignoring cultural values, including historic roads and facilities. Greenery did not have any designated functions	– Mainly related to security, the possibility of living in Warsaw and access to the labour market		– Wastelands – Gardens – Ruins – Buildings
**1982/2006** Perspective General Plan of Spatial Development of Warsaw	The decline of the socialist system and the last years of a strong position of spatial planning. The master plans drawn up at that time were not reflected in real investments	**Late modernism, technocracy**, strictly designated areas (wedges) of greenery with an assigned function, consistent with the state of development at the time, although insufficiently regulated in terms of planning	– The need for access to the areas of organised formal recreation—leisure parks and informal—employee allotment gardens		– Public park (Pole Mokotowskie) – Employee gardens – Cemetery and mausoleum of Soviet soldiers – Wastelands
**2006/2021** Study of the Conditions and Directions of Spatial Development of Warsaw (General Plan) Warsaw, 2006	**Liberal democracy** and the primacy of the free market, together with the weakness of the planning system, meant that the city has been unable to prepare a master plan for 15 years. Investment pressures, appropriation of public space, and local government's financial weakness make it difficult to plan and implement public investments, including green areas. The only greenery legally protected by special regulations is kept, the land is privatised	The absence of the urban composition emphasises the role of the designated green wedge in ventilating the city and regenerating air	– The need for access to meeting places in open areas, to gardens and water, to a network of bicycle and pedestrian routes connected with greenery – An increase of the ecological awareness (of a part of the society) and waiting for solutions increasing the city's resilience		– Public park (Pole Mokotowskie) –Employee allotment gardens – Cemetery-mausoleum for Soviet soldiers – housing estates

**Fig. 3:**
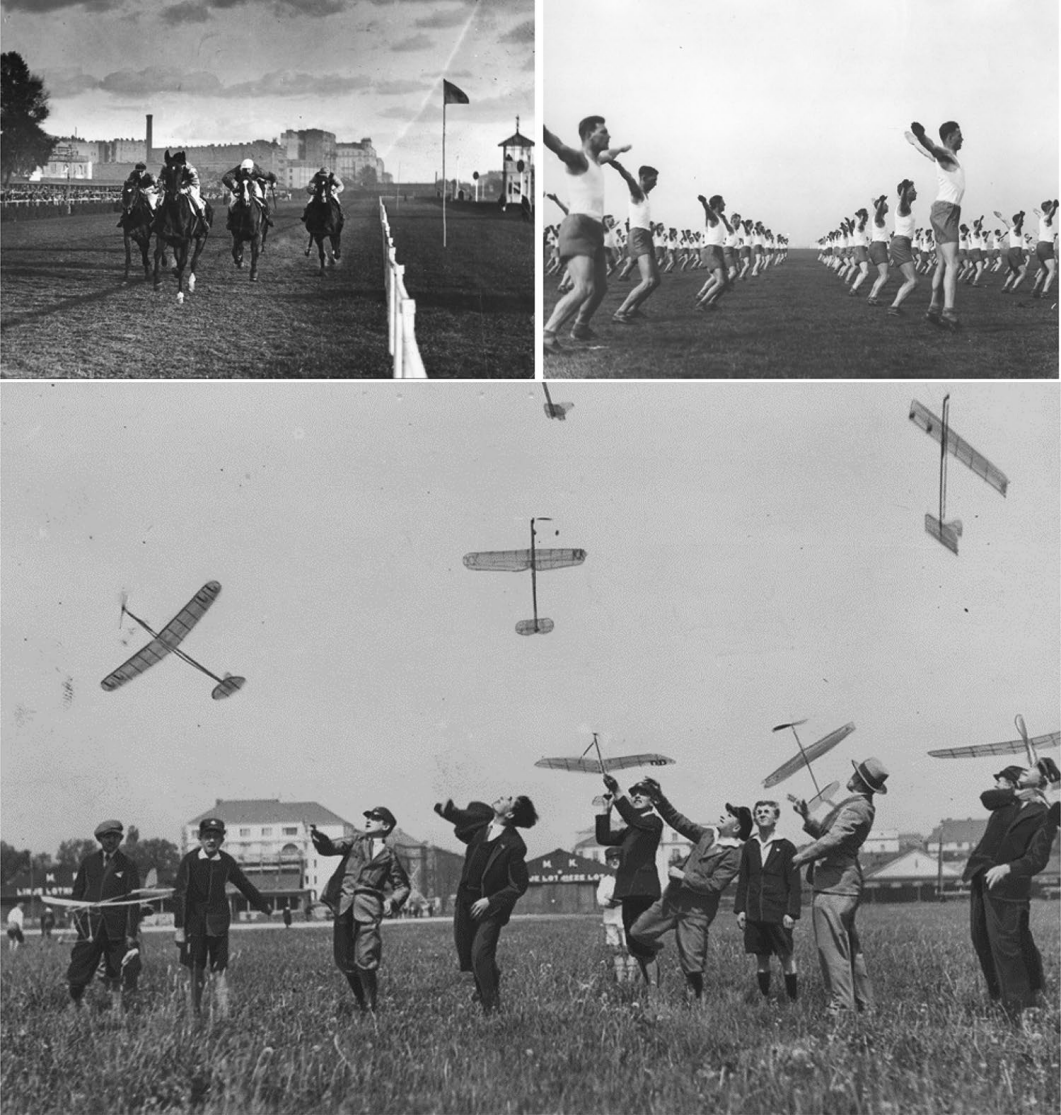
Various activities in open areas owned by the state, located almost in the centre of Warsaw—currently: Pole Mokotowskie Park. *Source* www.szukajwarchiwach.gov.pl, National Digital Archives—Poland, in the public domain

The first two spatial plans from the twentieth century were developed for a city with a smaller area than in the period after World War II (Fig. 2A, B). In line with the ideas of the early modernism, new development areas were indicated, but also some green areas that were to remain undeveloped. The plan from 1916 clearly demonstrated the idea of shaping the system of greenery, which consisted in connecting green alleys between larger and smaller green areas for various purposes in such a way as to form a system (Szmelter and Zdunek-Wielgołąska 2020). An important element of the plan were issues of hygiene and health, which can be considered a sign of modernity. The part of the studied strip of land, located closest to the city centre, served then as a horse racing track, airport and field for military exercises (Fig. 3). The plan indicated new districts and new green areas in their place (Fig. 2A).

**Fig. 4:**
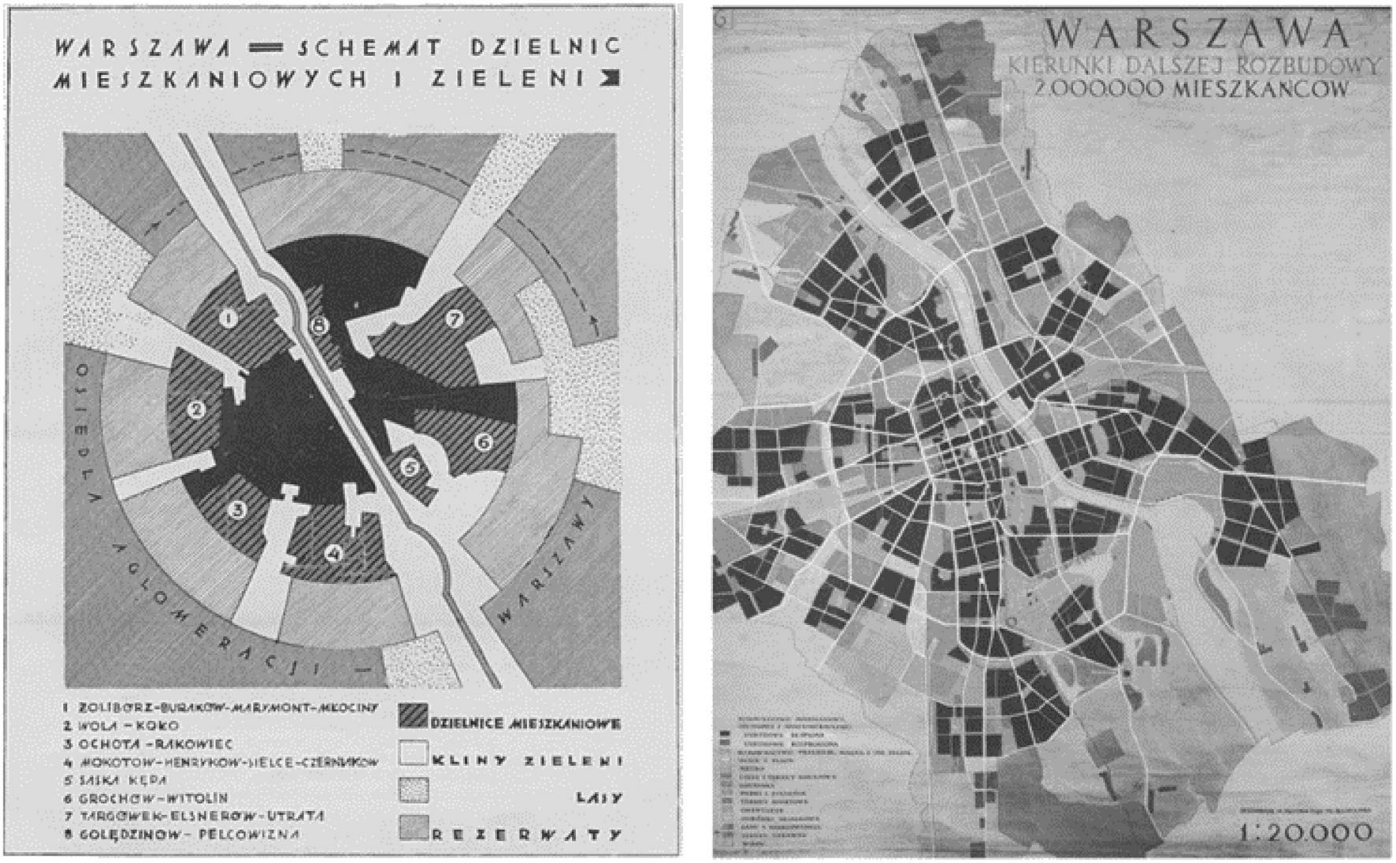
Plans of Warsaw. **A** On the left: diagram of residential districts and greenery, Warsaw, 1928. *Source* Buckiewiczówna (1928, p. 431). **B** On the right: General Plan of Warsaw. Directions for further expansion, 1954. *Source* Chmielewski (2004, p. 105)

Somewhat later, in the draft of the general plan of Warsaw, prepared in 1928, a vision of the radial-lane model of the capital and its surroundings was presented, and finally adopted in similar way in 1931 (Fig. 4A). Green belts were separated, structuring the entire spatial arrangement, a dense network of parks was introduced, and internal green belts added, placing this plan in early modernism, connected with the culturalist trend. The main reason for such a structure of the city was the care for the health of its inhabitants. In later years, it became the starting point for thinking about the lane-radial settlement system of Warsaw and its surroundings (Szmelter and Zdunek-Wielgołąska 2020). A wide wedge of greenery located in the study area was planned in previously undeveloped areas, or those that were de facto open areas with other functions (such as the airport and race track mentioned above). This inspired the creation of a southern corridor, later preserved in other spatial plans (Fig. 2B). It is worth noting, however, that this concept was not related to any special natural conditions. The only exception was the wedge along the Vistula Valley.

**Fig. 5:**
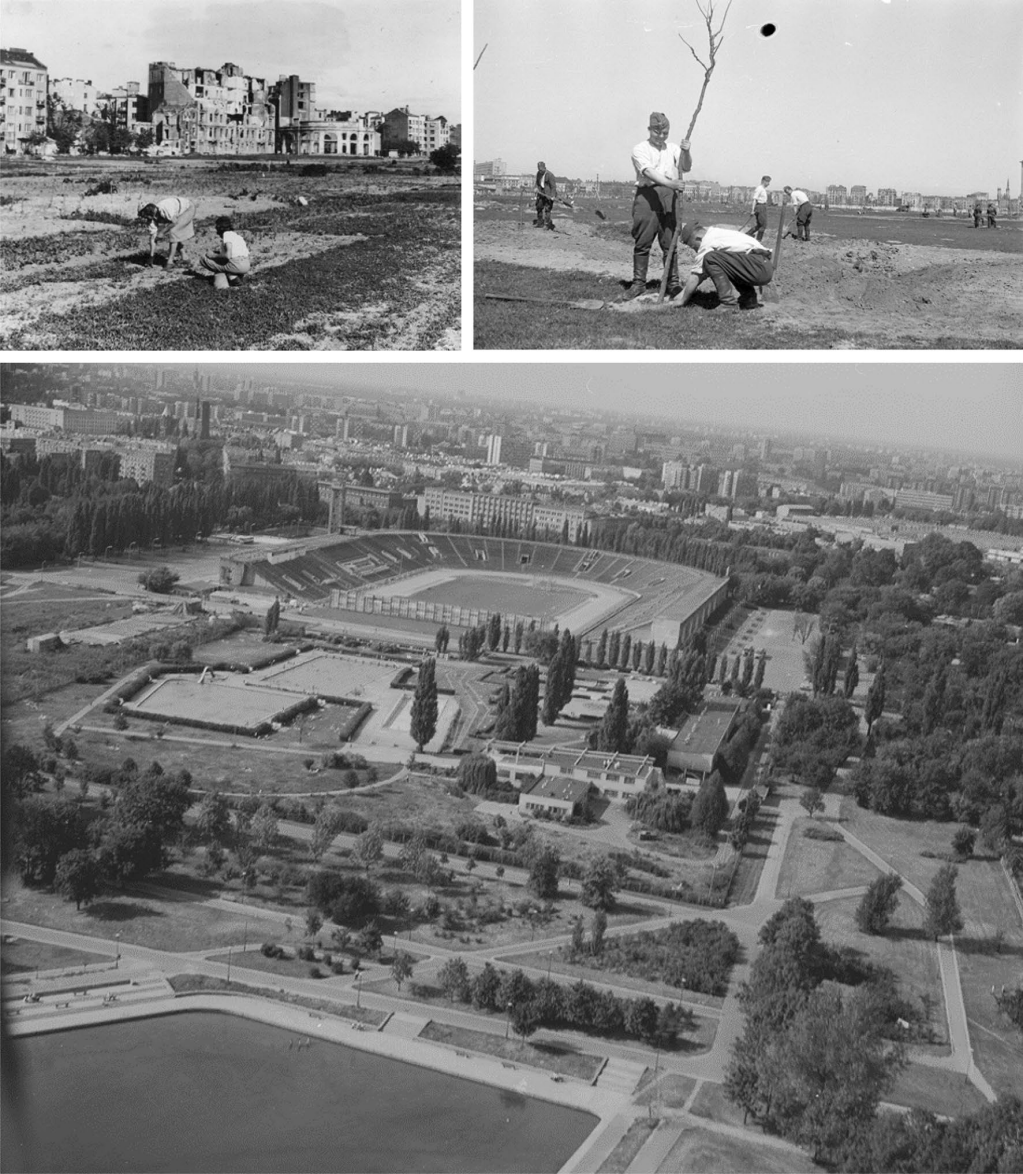
The area of the present Pole Mokotowskie Park. Top left—spontaneously established allotments in 1946, *Source* https://fotopolska.eu/377499,foto.html. Top right: tree planting action in the new park, 1948, *Source*: www.szukajwarchiwach.gov.pl, National Digital Archives—Poland, author: S. Rassalski. Below: the Skra Sports and Recreation Complex, *Source* www.szukajwarchiwach.gov.pl, National Digital Archives—Poland, in the public domain

The ideas of the avant-garde modern movement found fertile ground in Warsaw when it had to be rebuilt after the almost complete destruction and depopulation during the Warsaw Uprising in 1944 and the end of World War II. It contributed to adopting plans envisaging a highly loosened, multi-lane development, separated by extensive green areas. Such a layout was first presented in the city development concept from 1945 and later in the plans from 1946 and 1954 (Fig. 4B). Open areas structured the city space and, interestingly, exceeded the size of built-up areas. However, they did not form clear wedges but rather a grid of wide north–south stripes and narrow east–west passes, surrounded by a ring of greenery. Functionally undefined green areas were planned at the area of our research more or less where the pre-war plan indicated a green wedge, but much wider and further south, over the border of enlarged Warsaw. It was not difficult for the authorities to designate such areas, as the land in Warsaw was communalised. In the first years after World War II, vast open areas in the studied south belt were spontaneously used as allotments. A dozen or so years later, the planned development of urban open areas in this area began—a public park was established and sports centres were built (Figs. 2C, 5).

The wedge-shaped system of open areas, adopted in the general plan of Warsaw from 1931, became the basis for the system of ventilation corridors in later studies. Since the green wedges were mainly undeveloped zones and the emphasis was on rebuilding the central areas, they were kept—albeit in a reduced size—in subsequent plans, restoring them to their former function of ‘green lungs of the city’ and justifying it mainly on health grounds (Fig. 1). The studied southern corridor was one of the basic elements of this system (Fig. 2D). After many changes, this concept was transformed into the ‘Air Exchange and Regeneration System’, adopted in the General Plans of Warsaw from 1982 and 1992. And it was a great breakthrough in the approach to green wedges, which is also shown by the further transformations of the area we analysed in detail. These were no longer green areas excluded from development, as predicted by the earlier green belts concepts, but the lanes of land on which non-burdensome development was allowed—for their main function was to ventilate the city. These plans were very imprecise and quite ambiguously determined the green wedges and the rules for development. At the same time, it was the beginning of a new socio-political system in Poland. Thus, with a liberal economy and a free market expansion, many valuable green areas were lost and uncontrolled investments began to disturb all the greenery system. In the study area, the greatest success of the government was the creation of the public park, Pole Mokotowskie, at the turn of the 1970s and 1980s.

Today, the document in force is the general plan: ‘Study of the conditions and directions of spatial development’ of Warsaw, adopted in 2006. It indicates nine of the so-called ‘areas of air exchange and regeneration corridors’, distinguished within the ‘Warsaw Natural System’ areas. The rigours and restrictions for new investments are not particularly severe and do not in any way translate into guidelines for urban design. Therefore, in many air exchange corridors, new investments have been implemented in a manner inconsistent with the former idea of green belts. This is clearly shown by the fact that a significant part of the former southern wedge we are examining has been converted into a housing estate (Fig. 2E). Other wedges have also been ‘mutilated’, and the way they function must be reconsidered.

A new general plan for Warsaw is being prepared and the works are nearing completion. Although many initial analyses have already been carried out, the issue of green wedges is still being discussed, and expert opinions on their function are unsatisfactory. The function of ventilation is being questioned more and more often, as its scope has never been thoroughly investigated. Other functions, which were previously thought to be performed by the green wedges, seem to have been forgotten. In the discussions about the future of Warsaw, more and more is being said about the role of green infrastructure, not always defining it precisely, and at the same time the remnants of green wedges are being ignored more and more clearly. Spatial planning in Warsaw is the responsibility of its authorities, much depends on what arguments for maintaining and regenerating green wedges will be taken into account. Will the arguments of experts, investors or residents prevail? Being aware of the power of the voice of the local community in this discussion, we examined the opinions of residents about urban greenery, especially in relation to the green wedge under study.

### The current social approach to greenery and recreation in Warsaw

Social expectations differ not only depending on age groups, ownership status, but also on other experiences inherited after the transformation. In Poland, some groups downplay the real threats resulting from the deteriorating state of the urban environment and the consequences of climate change.

Although the social participation in planning processes is increasing, the current procedures underestimate the relationship between the city-building culture arising from past experiences and the expectations of various social groups towards nature and the ongoing changes. In this context, it is justified to focus on the extreme attitudes. The first one is that nature is an object to satisfy human needs, and the second that nature is an entity for which man is co-responsible. These different attitudes translate into a variety of expectations towards urban design and especially the green infrastructure programming process. A mismatch of expectations is particularly evident in societies touched by political transformation, where private interests tend to dominate over common good, leading to the further polarisation of the society. The awareness that the inhabitants need green areas is still not common among the authorities of cities and communes, which results in concrete-based investments in many public spaces, and therefore related to hardening the terrain and limiting infiltration-active areas (Mencwel 2020). On the other hand, the voice of residents and organisations is not always uniform, and sometimes the needs and expectations are divergent or even mutually exclusive (Domaradzka 2021).

In the case of Warsaw, an essential factor influencing the preferences towards green space is, for example, the place where residents lived before moving to the capital. Due to the destruction of the city during the Second World War, only a very small percentage of residents can track their roots to Warsaw for more than a generation back. As a vibrant economic centre, offering the best education and employment opportunities, Warsaw is also famous for being the city of internal immigrants—only around half of its adult population consists of people born in Warsaw. In the mid-1990s, during the time of the greatest free market boom, public opinion polls showed that the preference of ‘new Varsovians’ was to live as close to the city centre as possible, but at the same time surrounded by forest greenery and rural silence. Thirty years later, those preferences mainly remain valid for some residents. However, a recently noticed tendency is the return of young people to the city centre—house with a garden is no longer a dream. Instead, an apartment in the very centre of the city is the goal for many young people who switch to bikes and public transport and enjoy walkable and bikeable public green areas. Some of those new urbanites (Kubicki 2020) focus on access to recreational green areas, others engage in grassroots activities to create or revive green spaces in the city centre. In the last decade, community gardens, urban farms, green revitalisation of urban backyards and inner courtyards became part of a Warsaw’s middle class lifestyle. While older generations focus on cultivating the public allotment gardens, which remain accessible to the few, teenagers prefer open greenery that allows for urban recreation, including skateparks, public gyms or meeting places. Parents of small children are mostly interested in accessible parks with playgrounds and safe biking space for kids, while senior residents seek Nordic walking paths and comfortable sitting places in a safe and aesthetic environment.

According to the regular studies conducted by the City of Warsaw (Barometr Warszawski 2010–2021), urban greenery remains one of the aspects that most of Warsaw residents perceive as attractive and accessible. Growing citizen involvement in such projects is not surprising given as the Poles focus on greenery as a quality of life priority. Respondents of the recent national survey (PBS 2020) asked what the essential object in their neighbourhood is, indicated parks and other forms of urban greenery with recreational and relaxing functions more frequently (59%) than other public institutions. Nearly all respondents (97%) stated that it is important to have green areas in the vicinity, and for 84% this issue was perceived as very important. Significantly, 72% of Polish respondents believed that they have many green areas in the neighbourhood. This opinion is most often shared by people living in the countryside (82%), but inhabitants of big urban centres often reported they have a park within walking distance. As a result, nine out of ten respondents visited green areas in the vicinity of their residence at least twice a month in the last year, while 36% of the respondents did it every day. As many as 85% of Poles reported that they have the nearest green area within 10 min walk, while 56% lived only 5 min away from green areas. Importantly, the majority of Polish residents (83%) were satisfied with the quality of green areas in their neighbourhood.

When analysing those indicators from the perspective of socio-demographic characteristics, it can be observed that a park or other urban greenery was more often indicated as a priority by younger residents (aged 15–24: 68%, 25–34: 67%), living in large cities with more than 500,000 inhabitants (76%) who have the status of a student or school pupil (79%). This seems highly relevant for Warsaw, which has a large population of students and young adults interested in healthy lifestyle, searching for recreation opportunities and eager to enjoy green and clean environment.

Among the most frequently mentioned functions that the neighbourhood greenery should fulfil, the respondents mentioned the recreational and sports opportunities (38%), providing a place for rest and relaxation (37%), aesthetics, cleanliness (22%), walking paths (16%), fresh air (15%), trees, diverse vegetation (13%) and spaces for children (11%). A surprisingly high number of respondents (3/4) declared that they were ready to get involved in creating new and caring for the existing green areas in their neighbourhood.

To verify the socio-economic factors influencing Warsaw residents’ satisfaction with green areas, we analysed data from 2019 study conducted by the City of Warsaw Office ‘Quality of life of Warsaw districts’ residents’. We analysed the correlation between residents’ satisfaction with green areas and their social status at three levels: representative sample of Warsaw residents (*N* = 9720), representative sample of Mokotów district residents (*N* = 540) and the subsample of residents from 5 selected MSIs covering the area of Mokotów southern air exchange corridor (*N* = 380). We used the Pearson’s correlation analysis which is a common statistical tool for describing simple relationships without making a statement about cause and effect. It allowed us to measure the extent to which our variables were linearly related, reflecting the strength and direction of the association between them. Positive correlation values presented in the tables below means that both variables change in the same direction. A negative correlation means that the variables change in opposite directions. The higher the value of the correlation, the stronger is the dependence between values.

Our first analysis concerned the level of the whole City of Warsaw (Table 3). We discovered several aspects that correlated with lower satisfaction with green areas in the vicinity of where the respondent lived. First, people having children were more critical towards green spaces, while childless people evaluated green spaces more positively. Second, the better was the respondent’s household economic situation, the lower was his/her evaluation of green spaces. On the other hand, the general satisfaction with living in a certain part of the city correlated with a positive evaluation of green areas in the vicinity. Also, people with higher educational level as well as those who lived in the certain area longer were more positively inclined in their evaluation of neighbourhood’s green areas. While the relations discovered on the city level were not very strong, all the results were statistically significant.

**Table 3:** The correlation between residents’ satisfaction with green areas and their social status—Warsaw

Warsaw: city level	Having children	Satisfaction of living in the neighbourhood	The economic status of the household	Education level	Period of living in the district
Amount of greenery in the **area**	− 0.048*	0.122*	− 0.067*	0.009	0.080*
State of greenery in the **area**	− 0.069*	0.271*	− 0.056*	0.097*	0.101*
Amount and state of greenery in the **area**	− 0.074*	0.261*	− 0.085*	0.076*	0.111*
Amount of greenery in the **district**	− 0.066*	0.227*	− 0.042*	0.070*	0.097*
State of greenery in the **district**	− 0.058*	0.256*	− 0.021	0.087*	0.080*
Amount and state of greenery in the **district**	− 0.071*	0.272*	− 0.038*	0.093*	0.095*

* in tables indicates a statistically significant result

Our second analysis concerned the district of Mokotów, where the southern air exchange corridor is located. As illustrated in Table 4, the general satisfaction with living in this part of the city was even stronger correlated with a positive evaluation of the quality of green areas. However, those who were more happy with living in their neighbourhood were also more critical concerning the amount of greenery, while they had better opinion about its state. Moreover, the higher the educational level of respondents, the lower was their evaluation of district green areas—both its quantity and quality. However, the length of residency seemed to be positively correlated with the perception of district green areas. Having children or the economic status had no significant relation.

**Table 4:** The correlation between residents’ satisfaction with green areas and their social status—Mokotów district

Mokotów: district level	Having children	Satisfaction of living in the neighbourhood	The economic status of the household	Education level	Period of living in the district
Amount of greenery in the **area**	0.069	− 0.133*	0.077	− 0.065	0.038
State of greenery in the **area**	0.038	0.409*	− 0.076	− 0.032	0.062
Amount and state of greenery in the **area**	0.069	0.185*	− 0.004	− 0.055	0.038
Amount of greenery in the **district**	0.041	0.419*	− 0.006	− 0.127*	0.215*
State of greenery in the **district**	0.062	0.393*	− 0.060	− 0.112*	0.187*
Amount and state of greenery in the **district**	0.051	0.444*	− 0.048	− 0.109*	0.212*

* in tables indicates a statistically significant result

Our third analysis was based on the data from five selected areas of the City Information System (MSI), covering the area of Mokotów’s southern air exchange corridor. As shown in Table 5, we observed a statistically significant positive correlation between satisfaction with the quality of the nearby green spaces and overall satisfaction with the place where in which they live. Also, the longer people lived in the area, the more satisfied with the green areas they seemed to be. However, the household’s economic situation was consistently negatively correlated with the evaluation of green areas in both the closest neighbourhood and the district. The rest of the results was not statistically significant due to the small sample size.

**Table 5:** The correlation between residents’ satisfaction with green areas and their social status—southern wedge

Chosen MSI in the southern air exchange corridor—local level	Having children	Satisfaction of living in the neighbourhood	The economic status of the household	Education level	Period of living in the district
Amount of greenery in the **area**	0.043	− 0.125*	− 0.062	− 0.084	0.059
State of greenery in the **area**	− 0.054	0.291*	− 0.158*	0.059	0.062
Amount and state of greenery in the **area**	− 0.008	0.113*	− 0.145*	− 0.023	0.072
Amount of greenery in the **district**	− 0.036	0.243*	− 0.078	0.045	0.133
State of greenery in the **district**	− 0.034	0.236*	− 0.124	0.007	0.151
Amount and state of greenery in the **district**	− 0.034	0.236*	− 0.124	0.007	0.151

* in tables indicates a statistically significant result

To summarise, the residents of neighbourhood surrounding the ‘green wedge’ of Mokotów’s southern air exchange corridor were often critical concerning the amount of greenery in their neighbourhood. From the one hand, their satisfaction with living in the neighbourhood was strongly correlated with positive evaluation of the quality of green areas, but at the same time it was tied with higher expectations concerning its amount. Increased expectations concerning the access to high-quality green areas seem to be related with both higher socio-economic status as well as having children. In this context, the shrinking of Mokotów’s green wedge should be perceived as an important threat to the quality of life of local residents, including those with higher purchasing power.

## Discussion

The conducted research has shown that the southern green wedge has undergone major transformations, which reflects the changing approaches to urban design and city planning, the needs and preferences of residents, and the legal conditions. These changes are clearly depicted in Table 1 and Fig. 2. The existing urban greenery systems are closer to the concepts of early modernism than to the later avant-garde of the modern movement. However, there is a constant tendency to narrow down and break continuity of green wedges. A huge impact on the degradation of the role of the green wedge was to rename it as a ventilating corridor in which development was allowed. It was overlapped by the weakness of the planning system and investment pressures to which the city authorities succumbed as a result of the liberal free market economy, widely accepted after the collapse of the previous political and economic system.

But, on the other hand, research has shown substantial interest of Warsaw residents in the access to greenery. The expectations of the local community in relation to the functions of urban green areas vary depending on age, family and socio-economic situation. The residents expect further increase in the amount of greenery, which could support the preservation of green wedges idea, as long as it is properly implemented in spatial planning and urban design. An interesting fact is that wealthier residents have higher expectations concerning the greenery than those less affluent and more rooted in a given neighbourhood. This means that if district wants to attract more middle class residents it should reconsider putting more emphasis on saving the existing green wedge remnants as well as further developing greenery in the area. To avoid green gentrification, however, this type of development should be based on increasing the area of open accessible parks and playgrounds, and reversing the process of fencing off the best green areas within new housing settlements.

It should be also taken into account that COVID-19 pandemic has visibly changed the attitude towards greenery, highlighting its direct connection with personal well-being. As a result, according to research from March 2021, the inhabitants of Warsaw expect even more greenery and pro-ecological activities from the city mayor. Therefore, it seems that we are in a moment of dynamic change when previous social divisions around the issue of greenery are blurring. Nowadays, everyone seems to be in favour of increasing the quantity and quality of green areas. However, if it means that certain amenities like parking space would have to be given up in order to implement this postulate, affected residents may be less enthusiastic. With the increased number of biking paths and the well-developed public communication system, we can nevertheless expect an increased acceptance towards sustainable development ideas, paired with grassroots pressure for securing access to high-quality green areas.

Combining the research results and conclusions from the review of the literature, future scenarios and chances of maintaining the green wedges of Warsaw can be considered within the discussion.

**Fig. 6:**
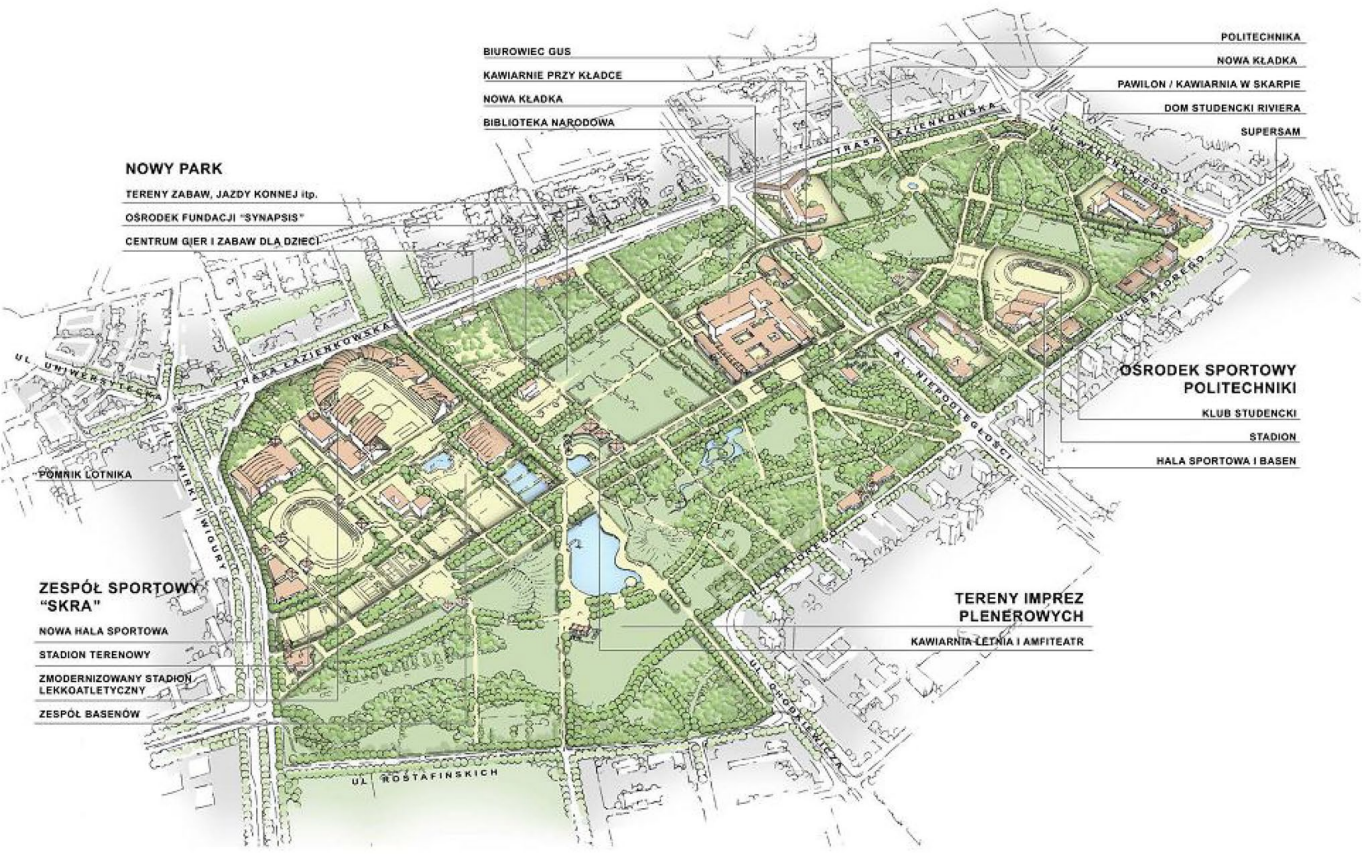
The concept of the development of the Pole Mokotowskie Park—the stage of preparation of the local spatial plan (Dawos, Krzysztof Domaradzki). *Source* Krzysztof Domaradzki Archive

**Fig. 7:**
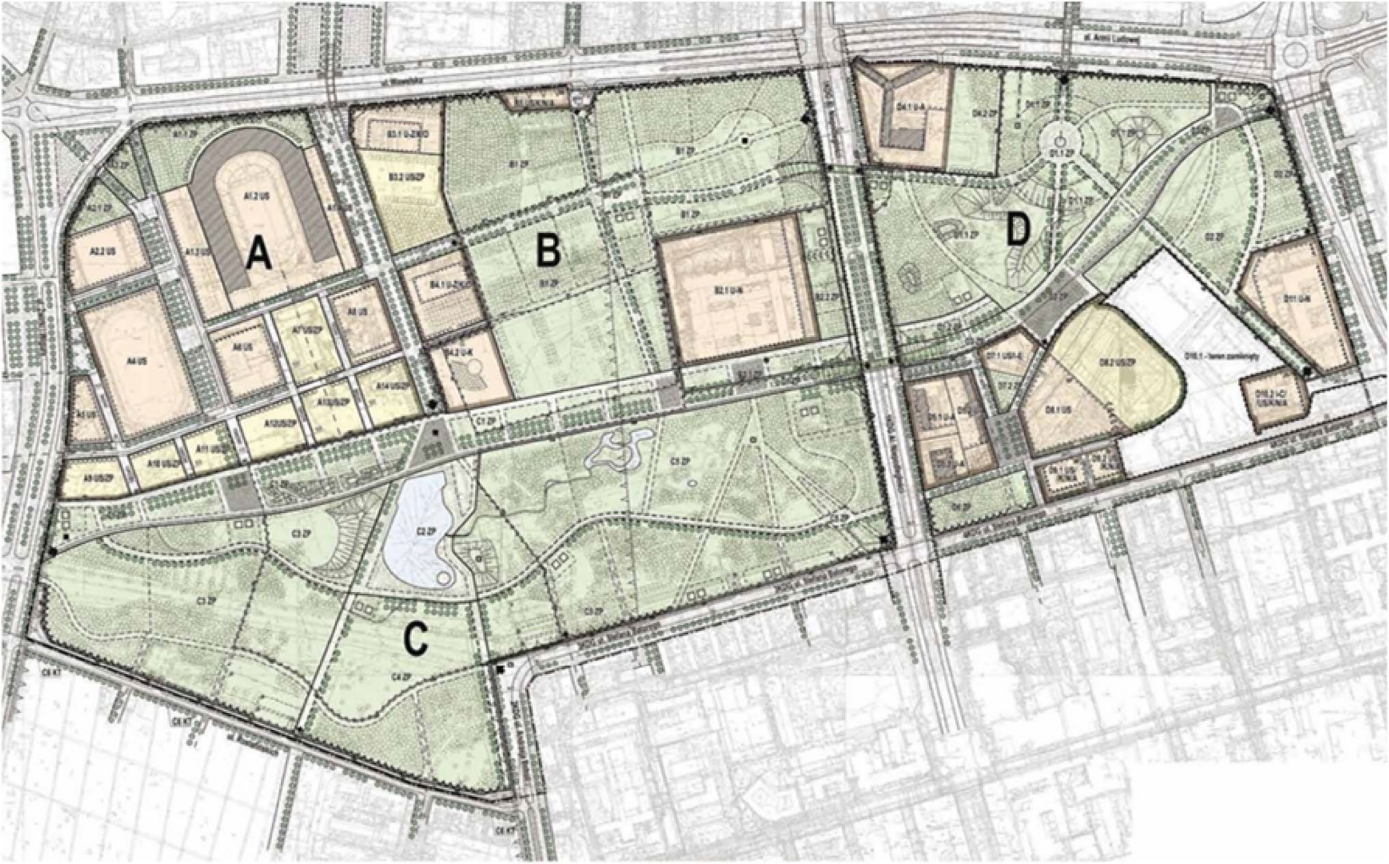
Map of the local spatial development plan of the Pole Mokotowskie Park. *Source* official website of the City of Warsaw (https://architektura.um.warszawa.pl/-/plany-obowiazujace-mokotow, accessed 10.12.2021)

**Fig. 8:**
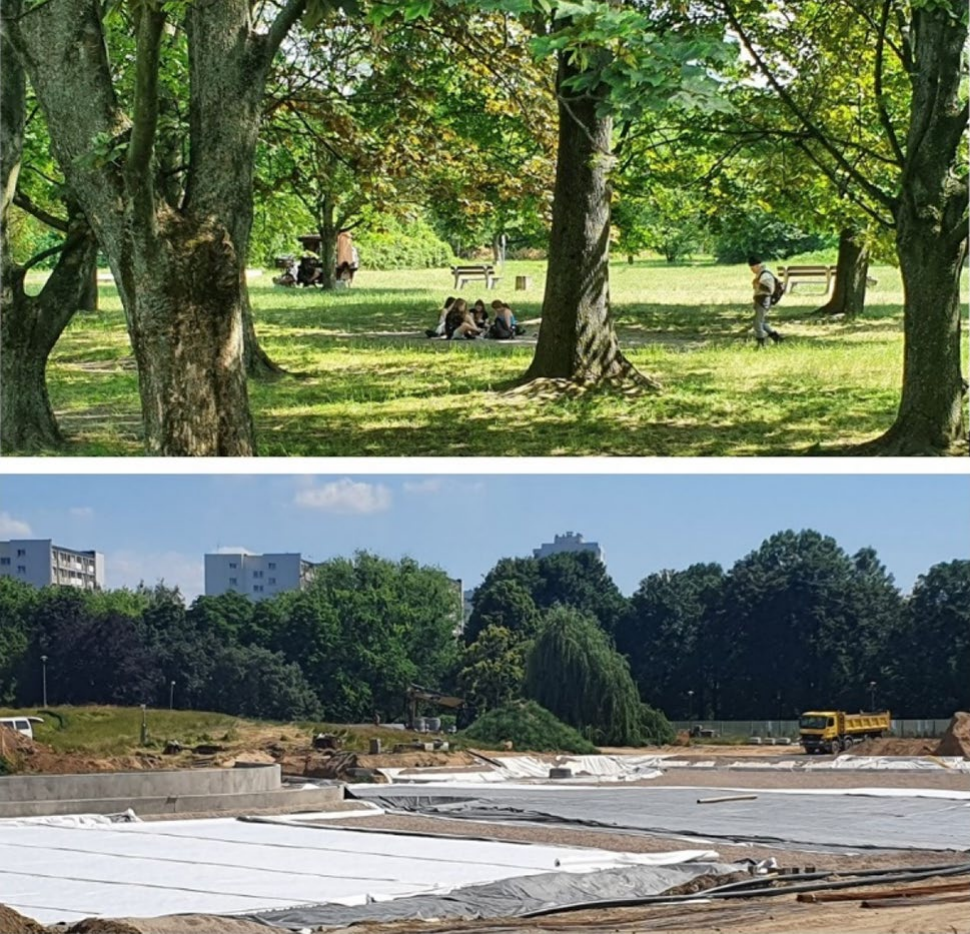
Currently, the Pole Mokotowskie Park is used for walks, picnics and games for children. At the same time, work is underway to modernise it and adapt it to the modern expectations of users. *Photo* K. Solarek

**Fig. 9:**
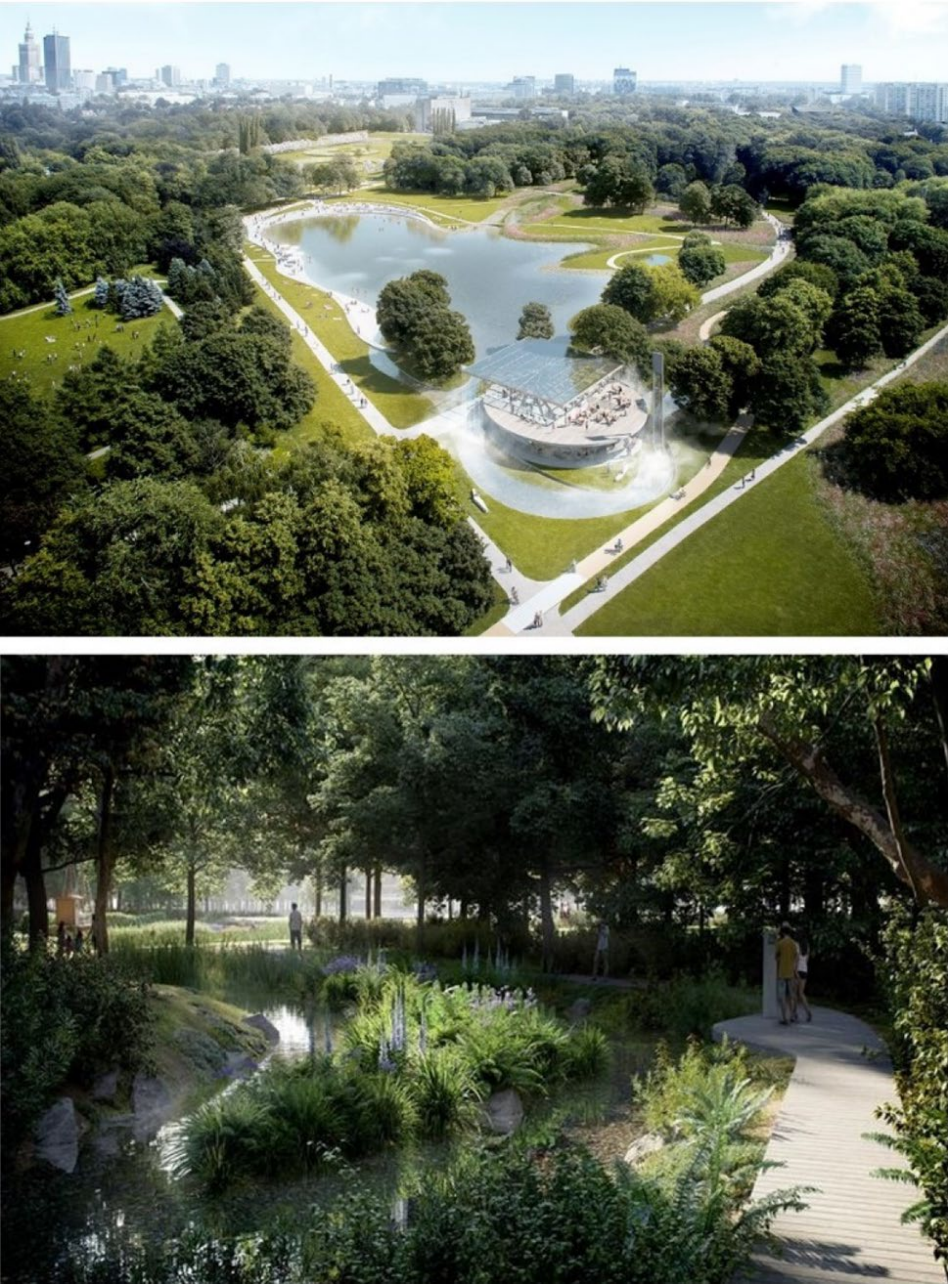
Visualisations of the Pole Mokotowskie transformation project towards more natural, pro-ecological solutions. *Authors* WXCA

The easiest, although not the cheapest, but also the most obvious step forward, is better protection of well-developed green areas with cultural values, such as, for example, public parks—in the case under study, the Pole Mokotowskie Park (Figs. 6, 7). This late modernist public park has been in existence for several years and the public interest has allowed for protecting its outskirts from development projects. Also, recently, social pressure has led to the conversion of concrete pools into natural ponds, the expansion and arrangement of new recreational places, the arrangement of a sensory and ‘biocenotic garden’, the construction of outdoor gyms and expansion of playgrounds, the construction of public toilets and introducing elements of small architecture (Figs. 8, 9).

Giving new meanings and functions to less spectacular fragments of urban greenery is much more difficult as it is challenged by various stakeholders. The most difficult challenge is to maintain green wedges on private properties. A new general plan for Warsaw is currently being prepared that may abandon this concept altogether. Already in the analysis of the existing state of development, carried out for the needs of the new plan, the lack of continuity of the wedges has been noted, and the idea of ventilating corridors is also criticised. On the other hand, the growing importance of sustainable water management and the enhancement of the role of green infrastructure is constantly underscored. However, the concept of green infrastructure is so broad that it can lead to the conversion of vast green areas into single investments with green roofs and walls. The complete abandonment of green wedges, which have been a permanent element of the city’s spatial structure for 100 years, would be an unfavourable change and a significant detriment to the identity, climate and inhabitants of the city. The hope for stopping this trend lies both in the European Green Deal Policy as well as an increased environmental awareness and growing expectations of Warsaw residents.

The presented social studies results, as well as the analysis of the changes in the city structure and the literature on the subject, lead us to consider what aspects should be taken into account when implementing spatial policy, including social dialogue in the context of planning green areas. This applies not only to Warsaw, but also to other cities with a similar history and urban structure.

### An anthropocentric versus eco-centric approach to the environment

A challenge for planning greenery systems relates to the existence of two attitudes towards nature in the environmental philosophy—the first one positioning humans at the centre, and the second treating them as an element of the ecosystem. In the case of Warsaw—the country’s capital with a hyper diverse population of 1.8 million—we encounter an increased polarisation between those anthropocentric and eco-centric approaches. Although the eco-centric narrative seems to dominate among the elites of Western countries, this does not mean that the views they represent are going to be assimilated among the opposition, who treat the natural environment as an object to be used. Especially since the anthropocentric attitude does not exclude concern for the natural environment (Kopnina et al. 2018), the assumption may be part of a policy of Green Deal (European Commission 2022).

In recent years in Poland, the leading role of the anthropocentric narrative could be observed. Therefore, its prevalence should not be ignored while shaping the policy concerning the green areas in the city. The patterns of sociocultural upbringing and education passed down from generation to generation remain the important determinants of social attitudes, the change of which may take even several generations. This means that the way of shaping urban greenery should also meet the expectations of both groups. Therefore, designing an inclusive policy in this field should be preceded by thorough research in this area and education focussed on shaping attitudes, to optimise future solutions and allow for social consensus in spatial planning.

### Understanding the place’s intrinsic value

Green heritage conveys the well-established communal ways of life, organised around specific values, customs and places. Over time, green areas acquire certain characters and functions that often become part of a local identity, stimulate place attachment and are meaningful to residents (Solarek and Grochowska 2021). It seems that such is, for example, the case of the green wedge being studied, or at least the part of it that has been available to the public for years. While some greenery becomes ‘petrified’ in its form and function, others remain a breeding ground for new activities and innovations. According to recent research (Roszczynska-Kurasinska et al. 2021), the social perception of changes concerning green spaces may depend on the qualities of places as well as communities and individual preferences. The expected effect of the change on the local well-being may be an important factor—some changes boost the comfort and happiness of communities in a balanced manner, others influence one aspect of life at the expense of another. Often change can be perceived as a trade-off between adding an attractive function and impairing the ‘spirit of the place’. Bearing this in mind, any green transformation should consider the long-term process that leads to its current meaning and social perception. The multifunctionality of urban green spaces is a challenge in itself. On the one hand, parks and gardens have an important recreational value and potential to influence people health and well-being in a direct way; on the other, they often have high aesthetic and historical value and remain monuments rather than accessible breathing spaces.

In the context of transforming the urban environment and adding new meanings or functions to the existing green sites, the notion of intrinsic value is crucial as it facilitates common meaning-making (Roszczynska-Kurasinska et al. 2019).

### Warsaw Baukultur of shaping green infrastructure

In Poland, there is an increased interest in promotion of national cultural achievements, therefore, it should be expected that the planning accomplishments in this area should also be appreciated, and become part of the process of building and strengthening local identities. We already know that the new paradigm of city planning must face the challenges of the present and the future, but also the experiences of the past. This does not mean, however, that the change in planning must be revolutionary and based on the negation of past achievements. Social studies suggest that a large part of Polish society does not expect radical changes and that Poles’ expectations are still deeply rooted in the past, which results in a greater attachment to traditional solutions, as well as the well-established ways of perceiving the world. The ‘Warsaw school of urbanism’ development plans for the capital were an expression of the spirit of the time. It is a city-building culture that has developed over the years and should be considered in future planning. This also applies to the concept of green wedges, so well crystallised in the past, which should not disappear in new spatial plans because they have both environmental as well as symbolic value. Therefore, the new vision should take into account the conditions and challenges as much as possible, while at the same time it should propose solutions that complement, reflect and repair the existing ones, in line with the building culture developed over the years in Warsaw.

## Conclusions

The conducted research showed how and why one of the most critical green belts in Warsaw changed from the era of modernism to the present day; most of the phenomena can be related to other elements of the urban greenery system in this city. We have also presented trends in a new approach to the functions of green areas, observed globally and locally, and social research has allowed for determining the needs of today’s city residents concerning green areas. Several conclusions can be drawn concerning the chances of permanently maintaining green belts within a sustainable city model envisioned in the new policy documents. And this may apply to other European cities where the legacy of modernism in urban greenery can be troublesome. The most important conclusions for various areas of urbanism—urban policy, urban planning and urban design are listed below.

### The urban policy context

Without effective tools for implementing spatial policy, the green heritage of modernism may be irretrievably lost. Urban greening need to fit in to the existing legal framework and to benefit from legal support and protection against competitive uses and destruction. However, much also depends on whether the local spatial policy will set itself one of the systemic goals of shaping green areas to move towards a sustainable city. There are many arguments for this to be the case. Sustainable development has become one of the main postulates of European integration, which in turn has been translated into the policies of individual countries and territories. Local spatial policy should consider residents’ preferences, but additionally, it should increase their awareness of the most critical urban values. Educating people and informing them about the environmental benefits of greenery, together with the recreational benefits, may be the motivating factors for public participation.

### The urban planning context

The troublesome legacy of the modern movement and the Charter of Athens postulates, that is, overestimated large green areas, often require changes. However, they should be conducted so as not to lose the essence of the planned systems, including green belts and wedges. To date, it remains one of the most effective tools for shaping urban structures. It is worth referring to the models of early modernism, which rationally justifies the division into built-up areas and areas excluded from development. It is likely that the course of some green bands will likely need to be revised, as they were sometimes marked using technocratic methods with no relation to soil, water and environmental conditions. Water management requirements or the enhancement of particular habitats can affect the corrections of these courses. In addition, the already-completed investments may also be a premise for such remodelling. For the critical areas of the city’s green areas to be permanently protected, development plans that prohibit building development are needed. Establishing in planning documents the course of venting corridors or green infrastructure networks—without prohibiting building development in the most important elements of the system—leads to the degradation of this system. Only good regulatory law protects natural resources for the common good regardless of whether they are on public or private land. The idea of green infrastructure represents an opportunity to re-appraise the green belts and wedges and, in some cases, to link them within a more extensive network of green infrastructure. However, areas for green infrastructure should be indicated on the plans’ drawings to include them in the city’s spatial structure permanently. The most challenging thing is to determine the destination—the functions of such areas that the greenery/green infrastructure could be preserved not only on public property lands.

### The urban design context

Urban and landscape design must consider both the need to preserve the identity of the place, its natural conditions, and new social needs and climate challenges. The devices and elements of the development of green areas should serve various users, be accessible for all, as well as connected with other public spaces. The increasing range of social expectations concerning the possibility of spending free time requires constant expansion of the programme offer. This, however, must not weaken the natural value of the places. Some green areas of the modernist era require revitalisation, including the reduction of paved areas, the naturalisation of reservoirs and water courses, and an increase in the scope of native and natural vegetation. The proportion of natural and artificial elements should be adapted to the primary function of a green area. Research has also shown that the aesthetics of green spaces can also be of great importance to many city dwellers, although it is not considered as important as meeting basic life needs.

## Data Availability

The data that support the findings presented in Table 3, 4 and 5 were made available by the City of Warsaw Office to Anna Domaradzka for the purpose of scientific publications. Data are available on request only from the City of Warsaw Office.
